# The Relationship Between Postpartum Depression and Timely Child Vaccination: A Systematic Review

**DOI:** 10.3390/vaccines13030222

**Published:** 2025-02-24

**Authors:** Julien Robitaille, Kayla Esser, Catherine King, Julie Leask, Kerrie Wiley, Simone Vigod, Gary Rodin, Shelly Bolotin, Gilla K. Shapiro

**Affiliations:** 1 Centre for Vaccine Preventable Disease, Dalla Lana School of Public Health, University of Toronto, Toronto, ON M5T 3M7, Canada; 2 School of Public Health, Sydney Infectious Diseases Institute, University of Sydney, Camperdown, NSW 2050, Australia; 3 Women’s College Research Institute, Women’s College Hospital, Toronto, ON M5G 1N8, Canada; 4 Department of Psychiatry, University of Toronto, Toronto, ON M5T 1R8, Canada; 5 Department of Supportive Care, Princess Margaret Cancer Centre, University Health Network, Toronto, ON M5G 2C1, Canada

**Keywords:** postpartum depression, preventative health, vaccination, systematic review

## Abstract

**Background/Objective:** Vaccines administered during early childhood rely on caregivers being aware, willing, and able to vaccinate their child. Postpartum depression (PPD) could adversely affect a parent’s ability to undertake such preventive care. This systematic review sought to examine the relationship between PPD and timely vaccination in children. **Methods:** We systematically searched eight databases (MEDLINE ALL, Embase, PsycINFO, CINAHL, LILACS, Web of Science, Sociological Abstracts, and Scopus) from database inception to September 2023. We also reviewed reference lists of included studies. We included primary studies that examined the association between PPD and child vaccination status between birth and 24 months. Two researchers independently extracted data and assessed study quality. **Results:** In total, 5504 records were screened for eligibility. Of the 50 articles included in full-text assessment, 12 met the eligibility criteria. Most studies (83%) were conducted in high-income countries, with a minority (17%) from lower-middle income countries (LMICs). The sample size of studies varied from <500 (33%) to >450,000 participants (17%). Overall, six studies (50%) found a relationship between maternal PPD and child vaccinations not completed on time, and six (50%) found no relationship. In most studies that were assessed to be of high-quality and found a relationship, the magnitude of the absolute risk was small. **Conclusions:** We detected significant heterogeneity among the included studies. Further high-quality research using standardized definitions is needed to determine whether parents with PPD may require tailored strategies and supports that consider their symptoms and specific barriers to vaccination.

## 1. Introduction

Vaccines are one of the most effective tools available to prevent morbidity and mortality of infectious diseases [1]. According to the World Health Organization (WHO), vaccines currently prevent 3.5 to 5 million deaths annually [2]. Many vaccines are administered during early childhood because this is a time when children are most likely to become seriously ill or die from an infection, they are first able to mount an effective immune response, and their immune responses are most robust [3]. Infants receive vaccines before age two to protect them from several infectious diseases, including measles, mumps, rubella, diphtheria, and others [3,4]. Immunization programs have also demonstrated cost-effectiveness, substantially reducing medical costs associated with vaccine-preventable diseases [5]. Children rely on their parents or guardians (hereafter parents) to consent to the administration of these vaccines, along with other necessary health care.

Postpartum depression (PPD) is characterized by clinically significant depressive symptoms lasting at least two weeks. PPD shares many symptoms of other types of depression including low mood, irritability, loss of interest or pleasure in things once enjoyed, lack of motivation, difficulty sleeping, changes in appetite, and feelings of hopelessness, guilt, and worthlessness [6,7]. PPD is often distinguished from the “baby blues”, which is a mild and transient mood disturbance occurring days after childbirth. There is no consensus on the time frame of the postpartum period and many research studies use time frames of up to one year [8,9]. The term “peripartum” onset has been introduced more recently to refer to a depressive episode with the onset of mood symptoms during pregnancy or in the four weeks following childbirth [7,10]. The change from describing postpartum to peripartum onset of depression occurred due to the recognition that 50% of postpartum major depressive episodes begin during pregnancy [10]. However, the emergence and trajectories of PPD symptoms can vary substantially, and, in a minority of individuals, elevated symptoms can persist for at least three years postpartum [11].

PPD, whether with elevated symptoms or meeting the full criteria for a mental disorder, affects about 15% of birthing parents, and about 5% of co-parents in the first postpartum year [12]. According to a recently published systematic survey and meta-analysis that included 291 studies in 56 countries, the prevalence of PPD is 17.7% [13]. PPD is reportedly greater in parents of children facing health challenges and stressful life events, and is influenced by social determinants of health [14,15,16,17]. Furthermore, according to a systematic review of 23 studies with data from 29,286 couples, both parents have been found to concurrently develop PPD in about 3% of couples [18]. Depressive symptoms may impact non-biological or non-carrying parents [19], consistent with the view that depression during the postpartum period is due not only to hormonal or physical effects of pregnancy but also to the psychological impact of the change in parenting status, identity, or caring for a newborn infant [20]. Depressive symptoms in new parents can be overlooked due to the emotional, psychological, and physical changes they experience [6,21]. For many parents, PPD remains untreated, as their symptoms are not screened or they do not seek out support [22].

PPD can have serious negative effects on the affected parent, their developing child, and the parent–child relationship. Studies have found that maternal depression has been associated with long-term poorer social/emotional, cognitive, language, motor, and adaptive behavior development in children [23]. PPD can also have wide-ranging impacts on childhood outcomes and health service utilization, including the use of preventive health care [17,24]. For example, children whose mothers had depressive symptoms at two to four months had reduced odds of receiving age-appropriate well-child visits at 12 months [25]. Yet, PPD has also been associated with increased nonroutine and emergency health visits, which can result in higher health care costs [26,27].

It is possible that PPD is associated with a less timely uptake of child vaccines. Parents with PPD of under-vaccinated infants may find it difficult to accomplish many routine activities of daily living, lacking the motivation or capacity to schedule, remember, and attend additional health appointments for their child. Depressive symptoms can impact cognitive functions and information processing [28], and parents with PPD may delay making decisions about vaccinating their child. In the literature, there has been mixed evidence regarding a potential association between parental PPD and timely vaccination in children, with some studies finding a negative association [29], and others finding no association [30]. The objective of this review is to systematically examine the relationship between PPD and the timeliness of child vaccination. Understanding this relationship is a first step towards determining whether health promotion interventions may be helpful to support child vaccine uptake among parents with PPD.

## 2. Methods

We conducted this systematic review in accordance with the Preferred Reporting Items for Systematic reviews and Meta-Analyses (PRISMA) guidelines (Supplementary Material S1) [31]. We preregistered our protocol in the International Prospective Register of Systematic Reviews (PROSPERO) on 24 November 2023, under registration number CRD42023482716 [32].

### 2.1. Search Strategy

An information specialist (C.K.) systematically searched the following eight electronic databases: MEDLINE ALL, Embase, PsycINFO, through OvidSP platform; Cumulative Index to Nursing and Allied Health Literature (CINAHL) via EBSCO; Latin American and Caribbean Health Sciences Literature (LILACS); Web of Science Core Collection; Sociological Abstracts; and Scopus. The search period was from database inception to September 2023. To minimize bias, we placed no limitations on date or language; we had access to translation. We included a combination of controlled vocabulary terms (where available) and text word terms to the search that related to PPD and child vaccination (see Supplementary Material S2), and we slightly adapted the search across each database to incorporate differing database search syntax. We also searched the reference lists of all included studies for additional studies.

### 2.2. Eligibility Criteria

We included studies that reported data from primary studies conducted with humans (e.g., cross-sectional, cohort, or case–control studies), were published in a peer-reviewed journal, and examined the association between parental PPD and child vaccination status between birth and 24 months. The timeliness of child vaccination status varied depending on the study setting and immunization program in each setting. Our definition of parents included carrying and non-carrying and biological and non-biological parents. Our definition of PPD encompassed peripartum depression (onset of mood symptoms during pregnancy or in the four weeks following delivery) [7,10], and, similar to previous research [8,9], we defined the postpartum period as the first 12 months and included all papers with a subset of parents during this timeframe. We included publications in any language, any low-, middle-, or high-income country setting, and all publication dates. 

We excluded studies from this review if they were non-human studies, studies without a control group, studies evaluating interventions, and qualitative studies. We excluded the grey literature, books and chapters, editorials, practice guidelines, abstract only reports, commentaries, and preprints. We also excluded records that did not have sufficient data to extract.

### 2.3. Study Selection and Extraction

Upon search completion, we removed duplicate records using reference management software (EndNote X9) [33]. We used Covidence software (https://www.covidence.org/) [34] for title and abstract screening and full-text screening. Two researchers screened the titles and abstracts of all records (J.R., K.E.). Disagreements were resolved in consultation with a third member of the study team (G.K.S.). For studies meeting the eligibility criteria, or those that were unclear from the abstract alone, the researchers then assessed the full text according to the inclusion/exclusion criteria, and accepted or rejected the paper as appropriate. Any uncertainty about the inclusion of a record was discussed with a subset of the research team (J.R., K.E., C.K., S.B., G.K.S.), who communicated regularly through e-mails and team meetings. 

We created and piloted a data extraction tool prior to conducting full extraction on five studies. Two reviewers (J.R., K.E.) independently extracted and verified the data on study characteristics using the standardized extraction form. We extracted data on the publication details (e.g., authors, year published, title, language); study details (e.g., objectives, design, data collection mode, year of data collection, country, site, setting); parent sample characteristics (eligibility criteria, sample size, pregnancy data, sex, gender, age, parenting composition); PPD details (definition, measure, clinical management of depression, postpartum psychosis); child vaccine details (vaccines examined, child age, child sex, receipt of vaccination, definition of timeliness); study findings (absolute risk, odds ratio, risk ratio, confidence intervals, χ-square, degrees of freedom, *p*-value, confounders); and study limitations as identified by the study authors. When studies were unclear about definitions or measures for either PPD or timeliness of vaccination, we requested this from the corresponding authors (up to three emails sent at intervals of one week apart). 

### 2.4. Quality Assessment 

Two researchers (J.R., K.E.) independently conducted a quality assessment and reconciled their assessments, with outstanding discrepancies being brought to additional members of the research team for resolution (S.B., G.K.S.). To evaluate the quality of each study, we applied the appropriate Joanna Briggs Institute (JBI) checklist tool [35], which was matched to study design (i.e., cross-sectional or cohort studies). Assessments were conducted at the study level. The JBI tool assessed the methodological quality of each study and determined the extent to which a study addressed the possibility of bias in its design, conduct, and analysis. 

### 2.5. Data Analysis and Synthesis 

We assessed for statistical heterogeneity in R version 4.2.1 using the I^2^ statistic to determine whether to conduct a meta-analysis (I^2^ < 50%) or narrative synthesis (I^2^ ≥ 50%) [32]. If a meta-analysis was deemed appropriate, we planned to conduct a fixed effect model. If a narrative synthesis [36] was deemed appropriate, we planned to summarize the evidence to highlight areas related to the study design and participant characteristics, with relevant tables, diagrams, and figures to illustrate results. 

## 3. Results

### 3.1. Search Results

**Figure 1:**
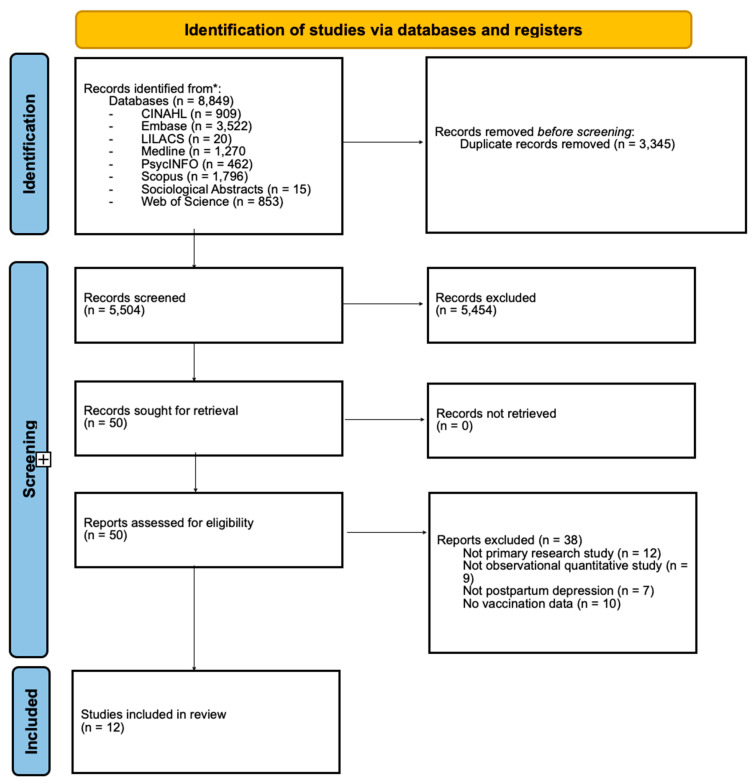
PRISMA flow diagram.

Figure 1 provides a PRISMA flow diagram detailing the study selection process. The database searches yielded 8849 records from eight databases. After removing 3345 duplicates, a total of 5504 individual records were screened to determine their eligibility based on title and abstract. Of those, 50 were identified as eligible for full-text review. Of the 50 full-text articles screened, 12 met the eligibility criteria and were included in the review. 

Studies were not suitable for pooling because of substantial heterogeneity in populations, outcomes (vaccines examined and timing of vaccination), and measures used to evaluate PPD. A narrative synthesis was therefore selected to summarize the search results and consider explanations for high heterogeneity between studies. The evidence synthesis focused on identifying whether an association between PPD and the timeliness of child vaccination was found, considering the definitions of PPD and timeliness of child vaccination that were used, along with determining other factors that may account for any differences between study findings. Accordingly, a χ-square analysis post-study registration [32] was conducted to examine whether there was a relationship between study sample size and whether the study found an association between PPD and child vaccination.

### 3.2. Study Characteristics

Table 1 provides a summary of the 12 studies included in this review. All studies were published in English between 1995 and 2023. Most studies (83%) were conducted in high-income countries, with a minority (17%) from lower/middle income countries. Studies were from several regions including the European (42%), American (33%), African (17%), and Western Pacific regions (8%). Most studies (92%) used a cohort design. The studies used multiple data collection methods, including a mixture of interviews (25%), questionnaires (83%), and health records (92%). The sample sizes of the studies varied from <500 to >450,000 participants, with a median sample size of 2724. All studies included only female parents (100%). To identify parental PPD, the majority (67%) of studies used a screening tool, while the remainder (33%) used medical records and prescriptions for antidepressants. Funding for the studies came from various, and often multiple, sources, including government (42%), university (42%), private foundations/organizations (42%), non-governmental organizations (8%), and vaccine manufacturers (8%); two studies (17%) did not report funding.

### 3.3. Quality Assessment

The methodological quality of the included studies is reported in Table 2 [37]. No studies were excluded following the quality appraisal process. The quality weightings of the papers were considered when reporting and discussing the results of the included studies.

Among the 11 cohort studies, 6 studies met all requirements of the JBI checklist [8,25,29,30,38,39]. The most consistent methodological limitation was an invalid or unreliable outcome measurement. The outcome was measured in a valid and reliable way for seven of the cohort studies [8,25,29,30,38,39,40]; however, three studies did not define the outcome in a valid and reliable way [41,42,43] and the outcome measure was unclear in one study [44]. The exposure was not measured in a valid and reliable way in one study [40]. Confounders were identified in nine cohort studies [8,25,29,38,39,40,41,42]; two studies did not identify confounders in their analysis [42,43]. Two studies also did not state strategies to deal with confounding factors [42,43].

One study had a cross-sectional design [45]. This study met all JBI checklist requirements, except the study outcome was not measured in a valid and reliable way [45].

**Table 2:** Quality assessment.

JBI: Cohort Studies Checklist	QA1	QA2	QA3	QA4	QA5	QA6	QA7	QA8	QA9	QA10	QA11
Farr, 2013 [38]	Yes	Yes	Yes	Yes	Yes	Yes	Yes	Yes	Yes	N/A	Yes
Lyngsøe, 2018 [39]	Yes	Yes	Yes	Yes	Yes	Yes	Yes	Yes	Yes	Yes	Yes
Matare, 2021 [41]	Yes	Unclear	Yes	Yes	Yes	Yes	No	Yes	Yes	N/A	Yes
Minkovitz, 2005 [25]	Yes	Yes	Yes	Yes	Yes	Yes	Yes	Yes	Yes	N/A	Yes
Osam, 2020 [29]	Yes	Yes	Yes	Yes	Yes	Yes	Yes	Yes	Yes	N/A	Yes
Ruohomäki, 2021 [42]	Yes	Yes	Yes	No	No	Yes	No	Yes	Yes	N/A	Yes
Smith, 2022 [30]	Yes	Yes	Yes	Yes	Yes	Yes	Yes	Yes	Yes	Yes	Yes
Turner, 2003 [44]	Yes	Yes	Yes	Yes	Yes	Yes	Unclear	Yes	Yes	N/A	Yes
Watson and Kemper, [40]	Yes	Yes	No	Yes	Yes	Yes	Yes	Yes	Yes	N/A	Yes
Zaikin, 2022 [8]	Yes	Yes	Yes	Yes	Yes	Yes	Yes	Yes	Yes	N/A	Yes
Zajicek-Farber, 2009 [43]	Yes	Yes	Yes	No	No	Yes	No	Yes	Yes	N/A	Yes
**JBI: Cross-Sectional Studies Checklist**	**QB1**	**QB2**	**QB3**	**QB4**	**QB5**	**QB6**	**QB7**	**QB8**			
Ndokera and MacArthur, 2010 [45]	Yes	Yes	Yes	Yes	Yes	Yes	No	Yes			

Abbreviations: QA1—Were the two groups similar and recruited from the same population? QA2 —Were the exposures measured similarly to assign people to both exposed and unexposed groups? QA3—Was the exposure measured in a valid and reliable way? QA4—Were confounding factors identified? QA5—Were strategies to deal with confounding factors stated? QA6—Were the groups/participants free of the outcome at the start of the study (or at the moment of exposure)? QA7—Were the outcomes measured in a valid and reliable way? QA8—Was the follow-up time reported and sufficient to be long enough for outcomes to occur? QA9—Was follow-up complete, and if not, were the reasons to loss to follow-up described and explored? QA10—Were strategies to address incomplete follow-up utilized? QA11—Was appropriate statistical analysis used? QB1—Were the criteria for inclusion in the sample clearly defined? QB2—Were the study subjects and the setting described in detail? QB3—Was the exposure measured in a valid and reliable way? QB4—Were objective standard criteria used for measurement of the condition? QB5—Were confounding factors identified? QB6—Were strategies to deal with confounding factors stated? QB7—Were the outcomes measured in a valid and reliable way? QB8—Was appropriate statistical analysis used? N/A—not applicable.

### 3.4. Relationship Between Postpartum Depression and the Timeliness of Child Vaccination

Table 3 describes the methods of each of the 12 included studies, and Table 4 describes the studies’ findings. Six studies (50%) found a significant negative association between PPD and the timeliness of child vaccination [25,29,39,41,43,44]. The study with the largest sample size in this review (*n* = 850,243), a retrospective cohort study conducted by Lyngsøe et al. (2018) in Denmark [39], found a 7% reduced likelihood of having completed diphtheria, tetanus, pertussis, and polio vaccination for children of mothers with recent depression (risk ratio (RR) 1.07, 95% confidence interval (CI) 1.04–1.10) and a 12% reduced likelihood for measles, mumps, and rubella (MMR) vaccination (RR 1.12, 95% CI 1.09–1.15). However, the vaccine uptake rates of children of depressed and non-depressed differed by ≤3%. Osam et al. (2020) [29] conducted a retrospective population-based cohort study with 479,949 mother–baby pairs in the United Kingdom and found a decreased likelihood of vaccination among two-year-old children of mothers with depression (adjusted odds ratio (aOR) 0.86, 95% CI 0.84–0.88). The difference in up-to-date vaccination was similar in mothers with depression (86.1%) and those without depression (88.0%). Notably, in a United States study with 4874 participants, Minkovitz et al. (2005) [25] reported a slightly larger (21%) reduced likelihood of vaccination rate among two-year-old children of mothers with depression (odds ratio (OR) 0.79, 95% CI 0.69–0.93). Up-to-date vaccination of children was 54.0% in mothers with depression and 61.9% in those without depression [25]. Two studies had smaller sample sizes. In a prospective cohort study in Australia with 159 participants, Turner et al. (2003) [44] found that children of mothers with depression were almost five times more likely to be vaccinated late or not at all (OR 4.92, 95% CI 1.39–17.39). Similarly, in a prospective cohort study with 134 participants, Zajicek-Farber (2009) [43] found a difference in proportions between mothers with depression who had completed their child’s vaccinations (60.8%) and mothers without depression (88.1%) in the United States (*X*^2^ *=* 12.77, *p* = 0.01).

The other six studies (50%) included in this review found no significant association between PPD and the timeliness of child vaccination [8,30,38,40,42,45]. Two large retrospective cohort studies with sample sizes of 196,329 in the United Kingdom and 24,263 in the United States, respectively, did not find an association between maternal PPD and the vaccination uptake of routine childhood vaccines [30,38]. Similarly, a prospective cohort study by Ruohomaki et al. (2021) [42] in Finland and a retrospective cohort study by Zaikin et al. (2022) [8] in Israel both had moderate sample sizes (*n =* 969 and *n =* 1390, respectively) and found no association. In addition, two studies with small sample sizes found no association, including a cross-sectional study with 278 participants in Zambia [45] and a prospective cohort study with 202 participants in the United States [40].

**Figure 2:**
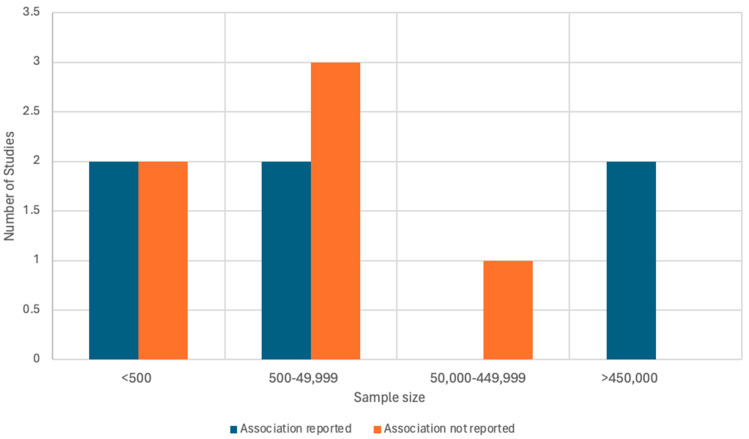
Comparison of studies by sample size. Note: We conducted a post-study registration examination to assess whether studies with larger sample sizes were more likely to find a significant association between PPD and child vaccination. No relationship was found between study sample size and whether the study found an association between PPD and child vaccination, χ-square = 3.2 (df = 3), *p* = 0.362.

Given that this review yielded mixed findings, with half of the studies finding a negative association and half finding no association, we conducted a post-study registration examination to assess whether larger study sample sizes were more likely to find a significant association. No relationship was found between study sample size and whether the study found an association between PPD and child vaccination, χ-square = 3.2 (df = 3), *p* = 0.362 (Figure 2). Differences between study findings were not explained by study setting, measurement of the exposure or outcome, confounders examined, or study quality. Confounders most often included sociodemographic factors related to the mother (e.g., age, income, and/or deprivation).

## 4. Discussion

To our knowledge, this is the first systematic review that has aimed to examine the relationship between PPD and timely vaccination in children. Overall, this review revealed a paucity of evidence regarding this relationship, identifying only 12 eligible studies. The findings on a potential association were mixed, with half of the studies reporting a negative association [25,29,39,41,43,44] and half reporting no association [8,30,38,40,42,45]. In most studies that found a relationship that were also assessed to be high-quality, the magnitude of the absolute risk was small [29,39]. An exception was one large American study that measured PPD using the Center for Epidemiologic Studies-Depression Scale and found low up-to-date vaccination rates in depressed (54.0%) and non-depressed (61.9%) mothers [25].

We sought to understand what factors may account for the mixed study findings. Most of the included studies (92%) were cohort studies (i.e., five were retrospective and six were prospective), while one study was cross-sectional. However, there was also remarkable heterogeneity among studies (e.g., by study setting, sample size, measurement of PPD or timely vaccination, confounders examined, or study quality), though this did not appear to explain differences found in study findings. For example, an association between PPD and child vaccination was found in two studies undertaken in the Americas [25,43], two in Europe [29,39], one in Africa [41], and one in the Western Pacific [44]. The sample sizes between studies ranged from less than 500 participants in four studies [40,43,44,45] to greater than 450,000 participants in two studies [29,39]. Given this substantial variation in study findings, we statistically evaluated whether larger studies were more likely to find an association between PPD and child vaccination, particularly given the relatively low rate of PPD and potential lack of power in small studies. However, we found no significant association between study sample size and study findings. 

This review revealed a lack of standardization in how PPD and the timeliness of child vaccination has been defined and measured, which made comparisons across included studies challenging. The exposure and outcome were not always precisely defined and varied substantially across studies. In future research, we recommend using validated tools, such as the Edinburgh Postnatal Depression Scale, which is considered to be the gold standard for PPD screening, along with using established clinical thresholds to enhance consistency and comparability [46]. There was also substantial variability in the definition of the PPD period between studies. Notably, while we intended to identify PPD in diverse parents, including those carrying (or not) and biological (or not), this was not possible. None of the studies in the review reported the gender of non-gestational or non-biological parents, and all studies reported PPD only in “mothers”. In addition, childhood vaccines and the timing of their administration were not uniformly specified, partly due to differing vaccination schedules across countries for both timelines and vaccine recommendations. Four studies did not specify which childhood vaccines were examined [41,42,43,45], which also made comparisons difficult. Furthermore, within our review’s inclusion criteria, there were variations in the child’s age range at the time that child vaccination was measured. It is critical that future research use standardized definitions of PPD and timely child vaccination.

The quality assessment of included studies indicated that the greatest limitations in the literature were outcomes not being measured in a valid and reliable way, and two studies did not examine known confounders. Commonly examined confounders included maternal age, income, and deprivation. Other potentially important covariates that were inadequately measured or not reported included depression symptom severity and duration, maladaptive behaviors associated with PPD, availability of social support, race or ethnicity, number of children in the family, household moves, and systemic immunization program policies. Indeed, contextual factors may influence whether PPD is associated with timely child vaccination. While some immunization programs provide systemic support (thereby reducing the reliance on parental initiative), other programs place a greater burden on parents to seek out vaccination, which may amplify the challenges posed by PPD. Future research should consider cultural attitudes and systemic differences when examining the relationship between PPD and vaccination behaviors. We recommend more rigorous assessment of relevant confounders in future research and greater consideration of the adjustment of multiple covariates that may also be associated with PPD. Future qualitative research could also help better understand what may account for a potential relationship between PPD and timely childhood vaccination and identify relevant covariates to further contextualize these findings. 

One mechanism that has been used to explain the association of PPD with several poor health outcomes for children [17] is a lower use of health services in parents with PPD [17]. However, if a relationship between PPD and timely childhood vaccination exists, the mechanism is likely not direct. New parents with PPD may either increase or decrease their use of health services for themselves and their infant depending on psychosocial factors such as attachment style and the parents’ coping mechanisms. Moreover, PPD may impact child vaccination outcomes through other drivers such as parental attitudes towards vaccines, ease of access to health care services, or their satisfaction with health services. For example, one study found that, despite mothers being satisfied with the attention their infants receive when accessing health care, some felt that their needs were being overlooked, and maternal depression was a predictor of lower satisfaction with child health care [47].

Recently, a prospective cohort study of 2762 Canadian women found that depression at less than 25 weeks of gestation and 4 months postpartum was not associated with a prolonged delay (i.e., delay for more than 6 months) of routine childhood vaccines [48]. While this study was outside of the scope of our systematic review’s study period, it identified several covariates that were associated with a prolonged delay in childhood vaccinations (i.e., income, household moves, Canadian born, and parity). The study’s conclusion that “health care providers can be reassured that maternal depression and anxiety do not appear to influence maternal commitment to routine immunization”, emphasizes the value of the multifaceted findings of this systematic review, while also highlighting the need for future research to delve deeper into the socioeconomic and demographic factors that may impact immunization in parents with PPD.

The findings of this review have several implications for clinical practice and the design of health services. PPD is a condition that has the potential to affect the uptake and timeliness of childhood vaccines, requiring tailored strategies and supports that take into consideration PPD symptoms and specific barriers to vaccination. Interventions to improve child vaccination coverage rates among parents with depression, including those with PPD, are available and can be integrated into a holistic health design. These interventions include a clear provider recommendation to vaccinate their child [49], motivational interviews during the perinatal or postnatal window [50], the inclusion of vaccination counselors or patient navigators to assist with supportive and logistic responsibilities [51], and reminder and recall systems [52,53]. There is currently limited integration of psychiatry and preventive services, including vaccination [54,55]. A greater emphasis on coordinating postpartum health services may support addressing low vaccination rates and untimely vaccination among children of patients with PPD. Home visits may be particularly important in such populations, where leaving the home is more challenging.

### Strengths and Limitations

This preregistered systematic review utilized robust methods to minimize bias, including a comprehensive search across eight databases with no date or language limits, and two independent reviewers for screening, extraction, and quality assessment. There were several limitations of the evidence reviewed and this review’s methodology. Most of the studies included in the review came from high-income countries, and only two studies came from low- and middle-income countries, limiting the generalizability of the study findings. This review also did not separately search the grey literature, which may have provided additional information on the potential association between PPD and the timeliness of child vaccination. However, some of the databases searched have embedded grey literature such as dissertations, conference papers, and working papers.

In addition, while the quality assessment tool used in this review was helpful in identifying whether there was a risk of bias, it was limited in examining each criterion in greater depth. For example, while the JBI tool identified whether confounders were included in analyses, it did not identify whether the included confounders were appropriate to measure. We also detected significant heterogeneity among the included studies (e.g., sample size, outcome definitions), which limited the ability to make direct comparisons. Lastly, the review was limited in scope to PPD, which focused solely on depression, and not on anxiety or other related psychosomatic symptoms during the postpartum period. Research has found an association between high levels of maternal anxiety (measured using the State-Trait Anxiety Inventory) and childhood vaccination coverage [56], suggesting that additional research is required to examine other psychological symptoms experienced by parents during the postpartum period. Specifically, we encourage future research to further investigate and synthesize the relationship between parental anxiety and timely child vaccination.

## 5. Conclusion

It is important to consider how to best support parents with PPD to maintain high vaccine coverage and timely child vaccination. This review highlights the overlooked role of PPD in potentially shaping childhood vaccine uptake. This study identified only 12 eligible studies. Study findings were mixed, with only half of the studies finding an association between PPD and timely child vaccination. We detected significant heterogeneity across the studies and inconsistent definitions and measurement of PPD and timely vaccination. More standardized research is needed to examine the association between PPD and the timeliness of childhood vaccination, and to identify what may account for the mixed findings uncovered in this review.

## Figures and Tables

**Table 1:** Study characteristics (*n* = 12 studies).

Characteristic	*n* (%)
**Publication Year**	
1995–2001	1 (8%)
2002–2008	2 (17%)
2009–2015	3 (25%)
2016–2023	6 (50%)
**Study Language**	
English	12 (100%)
Other	0 (0%)
**Country of Data Collection (by WHO Region)**	
African Region (AFR)	2 (17%)
Region of the Americas (AMR)	4 (33%)
Eastern Mediterranean Region (EMR)	0 (0%)
European Region (EUR)	5 (42%)
South-East Asian Region (SEAR)	0 (0%)
Western Pacific Region (WPR)	1 (8%)
**Country of Data Collection (by WBG Income Status)**	
High income (HIC)	10 (83%)
Upper/middle income (UMIC)	0 (0%)
Lower/middle income (LMIC)	2 (17%)
Low income (LIC)	0 (0%)
**Study Region**	
Local (county/district, hospital/institution, or city/village)	4 (33%)
Sub-national (state/province)	2 (17%)
National	6 (50%)
**Study Design**	
Cohort	11 (92%)
Cross-sectional	1 (8%)
**Data Collection Mode** ^1^	
Health records	11 (92%)
Interviews	3 (25%)
Questionnaires	10 (83%)
**Participant Sample Size**	
<500	4 (33%)
500–49,999	5 (41%)
50,000–449,999	1 (8%)
>450,000	2 (17%)
**Parent Sex (% female)** ^2^	
0–49%	0 (0%)
50–74%	0 (0%)
75–100%	12 (100%)
**Child Sex (% female)** ^2^	
0–49%	5 (42%)
50–74%	2 (17%)
75–100%	0 (0%)
Not reported	5 (42%)
**Source of Funding** ^1^	
Government	5 (42%)
University	5 (42%)
Private foundation/organization	5 (42%)
Non-governmental organization (NGO)	1 (8%)
Vaccine manufacturer	1 (8%)
Not reported	2 (17%)
None	0 (0%)
**PPD Identification**	
Medical records	4 (33%)
Use of screening tool	8 (67%)
Center for Epidemiologic Studies-Depression Scale (CES-D)	1 (12.5%)
Edinburgh Postnatal Depression Scale (EPDS)	4 (50%)
Self-Reporting Questionnaire-20 (SRQ-20)	1 (12.5%)
Duke Health Profile	1 (12.5%)
Rand Screening Instrument	1 (12.5%)
**Association Between PPD and Timeliness of Child Vaccination**	
No association	6 (50%)
Association	6 (50%)

^1^ Multiple categories could be selected; does not add up to 100%. ^2^ Gender identity was not recorded for any of the studies identified.

**Table 3:** Description of study methods.

References	Country, Study Region	Study Design	Data Collection Method, Date	Depression Definition	Timely Vaccination Definition	Sample Size (*n*)	**Vaccines Examined**
Farr, 2013 [38]	United States, sub-national	Retrospective cohort study	Electronic health records and questionnaires, 1998–2007	Depression during pregnancy and in the year after delivery, assessed using ICD-9 codes.	Received 3 doses of DTaP/DTP, 2 of Hep B, 3 of HiB, and 2 of Polio vaccine by 8 months old.	24,263	DTP; Hep B; HiB; Polio
Lyngsøe, 2018 [39]	Denmark, national	Retrospective cohort study	Electronic health records and questionnaires, 2000–2013	Depression during the 6 months before the childcare visit taking place after the child’s first year of life. Depression was assessed using hospital records and antidepressant prescription reimbursements.	Non-attendance to the recommended vaccination program for MMR (by 24 months), and DiTe (by 21 months).	850,243	DiTe; MMR
Matare, 2021 [41]	Zimbabwe, national	Prospective cohort study	Health records and surveys and interviews, 2012–2017	Depression symptoms assessed using the EPDS and/or suicidal ideation during baseline survey. Women were enrolled in study during pregnancy (at a median gestational age of 12.5 weeks); baseline was measured ~2 weeks after enrolment.	Not specified child immunization status (“fully immunized”) was transcribed from the child’s health records, or confirmed by the mother if the health records were not available.	4058	N/A
Minkovitz, 2005 [25]	United States, national	Prospective cohort study	Health records and surveys and interviews, 1996–2001	Depressive symptoms at 2–4 months after delivery, assessed using the CES-D.	Children were considered up-to-date if they had received 4 doses of DTP, 3 doses of Polio vaccine, and 1 dose of MMR by 24 months.	4874	DTP; Polio; MMR
Ndokera and MacArthur, 2010 [45]	Zambia, sub-national	Cross-sectional study	Electronic health records and questionnaires, date unclear	Depression between 2–12 months postpartum, assessed using the SRQ-20.	Not specified (“incomplete immunization refers to infants without all necessary immunizations for their age”).	278	N/A
Osam, 2020 [29]	United Kingdom, national	Retrospective cohort study	Electronic health records, 1993–2015	Maternal mental illness including depressive disorders between 1 year prior to birth and 2 years after delivery, assessed using ICD-10 codes.	Received 3 doses of 5-in-1, 1 of MMR by 24 months.	479,949	DTP, Polio, HiB (5-in-1); MMR
Ruohomäki, 2021 [42]	Finland, local	Prospective cohort study	Questionnaires, 2012–2017	Elevated depressive symptoms at eight weeks postpartum according to EPDS scores.	Not specified, “lacking regular vaccinations” assessed using a questionnaire for mothers based on self-report: “Has your child received all the regular vaccinations”.	969	N/A (“lacking regular vaccinations”)
Smith, 2022 [30]	United Kingdom, national	Retrospective cohort study	Electronic health records, 2006–2016	Depressive symptoms, postnatal depression, or an antidepressant diagnosis up to 1 year after delivery, according to medical records.	Received 3 doses of 5-in-1 vaccine by 12 months.	196,329	The 5-in-1 combined vaccination protects against DTP, polio, and HiB
Turner, 2003 [44]	Australia, local	Prospective cohort study	Electronic health records and questionnaires, 1990–1991	Depression at 7 months after delivery, according to the Duke Health Profile.	Received all required doses of DTP, OPV, and HiB at 2 months old, 4 months old, and 6 months old. Late staters or non-initiators were compared with timely maintainers.	159	DTP; OPV; HiB
Watson and Kemper, [40]	United States	Prospective cohort study	Electronic health records and questionnaires, 1990–1991	Depression during an undefined time period, according to the Rand screening instrument.	Received 4 doses of DTP, 3 doses of OPV, and 1 dose of MMR with no more than 2 months of delay between the recommended age and receipt of vaccination. Immunization records of children ≤24 months were reviewed for immunization delays.	202	DTP, OPV, MMR
Zaikin, 2022 [8]	Israel	Retrospective cohort study	Electronic health records and questionnaires, 2006–2019	Maternal PPD during the first 12 months after delivery, according to the EPDS.	(i) Received 1 dose of HBV between 28 and 55 days old. (ii) Received 1 dose of 1 of the following vaccinations: IPV, DTaP/DTP/DTAP, HiB, PCV13, Rota between 42 and 83 days. (iii) Received 3 doses of vaccine against pertussis (DTaP, DTP, DTAP + IPV DTAP + eIPV, DTaP + HiB, DTaP + HiB + IPV+ HBV) between 7 and 18 months. (iv) Received 4 doses of the quintuple vaccine (DTaP/DTP + IPV + HiB) by 18 months.	1390	HBV, IPV, DTaP/DTP/DTAP, HiB; PCV13; Rota
Zajicek-Farber, 2009 [43]	United States	Prospective, longitudinal cohort study	Electronic health records, interviews, and questionnaires, 2002–2006	Maternal depressive symptoms during the first 2, 6, and 16–18 months after delivery, assessed using the EPDS.	Completed all recommended immunizations by 18 months old.	134	N/A (“all immunizations”)

Abbreviations: CES-D—Center for Epidemiologic Studies Depression Scale; DiTe—diphtheria, tetanus, pertussis, and polio vaccine; DTaP—diphtheria, tetanus, and pertussis vaccine; DTP—diphtheria, tetanus, pertussis vaccine; eIPV—enhanced inactivated poliomyelitis vaccine; EPDS—Edinburgh Postnatal Depression Scale; HBV—hepatitis B vaccine; Hep B—hepatitis B vaccine; HiB—Haemophilus influenzae vaccine; ICD-9—International Statistical Classification of Diseases, 9th Revision; ICD-10—International Statistical Classification of Diseases, 10th Revision; IPV—inactivated poliomyelitis vaccine; MMR—measles, mumps, and rubella vaccine; mth—month; N/A—not available; OPV—oral poliomyelitis vaccine; PCV13—pneumococcal conjugate vaccine; PPD—postpartum depression; Rota—rotavirus vaccine; SRQ-20 = Self-Reporting Questionnaire-20.

**Table 4:** Description of Study Findings.

References	Vaccine Uptake Rates, *n* (%)	Confounders	Findings
Farr, 2013 [38]	Depressed = 2420 (74.3%) vs. not depressed = 16,079 (76.6%)	Adjusted for age of mother, education, marital status, Medicaid status, race/ethnicity, parity, hypertension/preeclampsia, and tobacco use.	No association was found between depression and receipt of all recommended immunizations, RR = 1.0 [1.0–1.0].
Lyngsøe, 2018 [39]	*MMR:* Depressed = 4594 (20%) vs. not depressed = 115,719 (17%) *DiTe:* Depressed = 4723 (23%) vs. not depressed = 153,965 (21%)	Adjusted for age of mother at birth, maternal education, maternal income decile, marital/cohabitation status, calendar year, paternal depression, maternal mental comorbidities, and parity.	Association was found between depression and non-attendance to the recommended vaccination program for MMR (RR = 1.12 [1.09–1.15]) and DiTe (RR = 1.07 [1.04–1.10]).
Matare, 2021 [41]	N/A	Adjusted for maternal age, education (years of completed schooling), and household wealth.	Association was found between depression and child fully immunized, aOR = 0.67 [0.50–0.90], *p* = 0.008.
Minkovitz, 2005 [25]	Depressed = 450 (54.0%) vs. not depressed = 2416 (61.9%)	Adjusted for mother’s age at child’s birth, race, ethnicity, marital status, education, mother employment status, parity, father’s employment status, child’s sex, household income, home ownership, child’s insurance, low birth weight, child’s health status, intervention status, and enrollment site.	Association was found between depression and up-to-date vaccination, OR = 0.79 [0.68, 0.93].
Ndokera and MacArthur, 2010 [45]	*Incomplete immunization*: Depressed = 6 (22.2%) vs. not depressed = 42 (16.7%)	Adjusted for infant age and location.	No association was found between depression and incomplete immunization, RR = 1.33 [0.62–2.83], *p* = 0.432.
Osam, 2020 [29]	Depressed = 67,241 (86.1%) vs. not depressed = 338,398 (88.0%)	Adjusted for child’s sex, ethnicity, delivery year, maternal age, practice level deprivation quintile, and region.	Association was found between depression and up-to-date vaccination, aOR = 0.86 [0.84–0.88]).
Ruohomäki, 2021 [42]	*Lacking regular vaccination:* Depressed = 7 (6.8%) vs. not depressed = 84 (9.9%)	No confounders.	No association was found, χ^2^ = 1.0, *p* = 0.314.
Smith, 2022 [30]	Depressed = 19,786 (95.1%) vs. not depressed = 165,691 (94.4%)	Adjusted for age and social deprivation.	No association was found, adjusted IRR = 1.01 [0.99–1.02].
Turner, 2003 [44]	N/A	Adjusted for mother’s age and income.	Association was found between depression and reduced timeliness of child vaccination, OR = 4.92 [1.39–17.39].
Watson and Kemper, [40]	*Delayed immunization:* Depressed (37%) vs. not depressed (36%)	Adjusted for child age, maternal age, and education.	No association was found.
Zaikin, 2022 [8]	(i) Depressed = 660 (93.1%) vs. not depressed = 633 (93.0%); (ii) Depressed = 667 (94.1%) vs. not depressed = 646 (94.9%); (iii) Depressed =500 (70.5%) vs. not depressed = 500 (73.4%); (iv) Depressed = 663 (93.5%) vs. not depressed = 627 (92.1%).	Maternal age, socioeconomic status, child’s gender, birth order of child, and child’s year of birth were used in matching mothers in the two groups.	No association was found between depression and vaccine uptake for: (i) RR = 0.98 [0.68–1.44], *p* = 0.919; (ii) RR = 1.15 [0.74–1.78], *p* = 0.523; (iii) RR = 1.11 [0.94–1.31], *p* = 0.229; (iii) RR = 0.82 [0.56–1.95], *p* = 0.229.
Zajicek-Farber, 2009 [43]	Depressed = 74 (60.8%) vs. not depressed = 60 (88.3%)	No confounders.	Association was found between depression and incomplete child immunization, χ^2^ = 12.77, *p* = 0.01.

Abbreviations: aOR—adjusted odds ratio; DiTe—diphtheria, tetanus, pertussis, and polio vaccine; IRR—incidence rate ratio; MMR—measles, mumps, and rubella vaccine; N/A—not available; OR—odds ratio; RR—risk ratio.

## Data Availability

The original contributions presented in this study are included in the article/Supplementary Material. Further inquiries can be directed to the corresponding author(s).

## Supplementary Materials

The following supporting information can be downloaded at: https://www.mdpi.com/article/10.3390/vaccines13030222/s1. Supplementary Material S1: PRISMA Checklist; Supplementary Material S2. Search Strategy.
