# Supplementary material for: The Relationship Between Postpartum Depression and Timely Child Vaccination: A Systematic Review
Source: Vaccines (Basel). 2025 Feb 24;13(3):222. doi: 10.3390/vaccines13030222 (PMC11946840; doi:10.3390/vaccines13030222)
Supplement: Supplementary file 1 [file vaccines-13-00222-s001.zip › vaccines-3411040-supplementary.pdf]

Supplementary Material S1. PRISMA Checklist

Section and Topic	Item #	Checklist item	Location where item is reported
**TITLE**			
Title	1	Identify the report as a systematic review.	Page 1, 3
**ABSTRACT**			
Abstract	2	See the PRISMA 2020 for Abstracts checklist.	Page 2
**INTRODUCTION**			
Rationale	3	Describe the rationale for the review in the context of existing knowledge.	Pages 3-5
Objectives	4	Provide an explicit statement of the objective(s) or question(s) the review addresses.	Page 5
**METHODS**			
Eligibility criteria	5	Specify the inclusion and exclusion criteria for the review and how studies were grouped for the syntheses.	Pages 6-7
Information sources	6	Specify all databases, registers, websites, organisations, reference lists and other sources searched or consulted to identify studies. Specify the date when each source was last searched or consulted.	Page 6
Search strategy	7	Present the full search strategies for all databases, registers and websites, including any filters and limits used.	Supplementary Material 2
Selection process	8	Specify the methods used to decide whether a study met the inclusion criteria of the review, including how many reviewers screened each record and each report retrieved, whether they worked independently, and if applicable, details of automation tools used in the process.	Pages 7-8
Data collection process	9	Specify the methods used to collect data from reports, including how many reviewers collected data from each report, whether they worked independently, any processes for obtaining or confirming data from study investigators, and if applicable, details of automation tools used in the process.	Pages 7-8
Data items	10a	List and define all outcomes for which data were sought. Specify whether all results that were compatible with each outcome domain in each study were sought (e.g. for all measures, time points, analyses), and if not, the methods used to decide which results to collect.	Pages 7-8
	10b	List and define all other variables for which data were sought (e.g. participant and intervention characteristics, funding sources). Describe any assumptions made about any missing or unclear information.	Pages 7-8
Study risk of bias assessment	11	Specify the methods used to assess risk of bias in the included studies, including details of the tool(s) used, how many reviewers assessed each study and whether they worked independently, and if applicable, details of automation tools used in the process.	Page 8
Effect measures	12	Specify for each outcome the effect measure(s) (e.g. risk ratio, mean difference) used in the synthesis or presentation of results.	Page 8
Synthesis methods	13a	Describe the processes used to decide which studies were eligible for each synthesis (e.g. tabulating the study intervention characteristics and comparing against the planned groups for each synthesis (item #5)).	Page 8
	13b	Describe any methods required to prepare the data for presentation or synthesis, such as handling of missing summary statistics, or data conversions.	Page 9
	13c	Describe any methods used to tabulate or visually display results of individual studies and syntheses.	Page 9
	13d	Describe any methods used to synthesize results and provide a rationale for the choice(s). If meta-analysis was performed, describe the model(s), method(s) to identify the presence and extent of statistical heterogeneity, and software package(s) used.	Page 9
	13e	Describe any methods used to explore possible causes of heterogeneity among study	Page 9

Section and Topic	Item #	Checklist item	Location where item is reported
		results (e.g. subgroup analysis, meta-regression).	
	13f	Describe any sensitivity analyses conducted to assess robustness of the synthesized results.	Page 9
Reporting bias assessment	14	Describe any methods used to assess risk of bias due to missing results in a synthesis (arising from reporting biases).	N/A
Certainty assessment	15	Describe any methods used to assess certainty (or confidence) in the body of evidence for an outcome.	N/A
**RESULTS**			
Study selection	16a	Describe the results of the search and selection process, from the number of records identified in the search to the number of studies included in the review, ideally using a flow diagram.	Pages 9-11, Figure 1
	16b	Cite studies that might appear to meet the inclusion criteria, but which were excluded, and explain why they were excluded.	N/A
Study characteristics	17	Cite each included study and present its characteristics.	Pages 12-14, Table 1
Risk of bias in studies	18	Present assessments of risk of bias for each included study.	Pages 15-17, Table 2
Results of individual studies	19	For all outcomes, present, for each study: (a) summary statistics for each group (where appropriate) and (b) an effect estimate and its precision (e.g. confidence/credible interval), ideally using structured tables or plots.	Page 18-24, Table 3
Results of syntheses	20a	For each synthesis, briefly summarise the characteristics and risk of bias among contributing studies.	Pages 18-19
	20b	Present results of all statistical syntheses conducted. If meta-analysis was done, present for each the summary estimate and its precision (e.g. confidence/credible interval) and measures of statistical heterogeneity. If comparing groups, describe the direction of the effect.	Pages 18-19
	20c	Present results of all investigations of possible causes of heterogeneity among study results.	N/A
	20d	Present results of all sensitivity analyses conducted to assess the robustness of the synthesized results.	Pages 19, 25 Figure 2
Reporting biases	21	Present assessments of risk of bias due to missing results (arising from reporting biases) for each synthesis assessed.	N/A
Certainty of evidence	22	Present assessments of certainty (or confidence) in the body of evidence for each outcome assessed.	N/A
**DISCUSSION**			
Discussion	23a	Provide a general interpretation of the results in the context of other evidence.	Pages 26-30
	23b	Discuss any limitations of the evidence included in the review.	Pages 26-30
	23c	Discuss any limitations of the review processes used.	Page 30
	23d	Discuss implications of the results for practice, policy, and future research.	Pages 29, 31
**OTHER INFORMATION**			
Registration and protocol	24a	Provide registration information for the review, including register name and registration number, or state that the review was not registered.	Page 5
	24b	Indicate where the review protocol can be accessed, or state that a protocol was not prepared.	Page 5
	24c	Describe and explain any amendments to information provided at registration or in the protocol.	N/A
Support	25	Describe sources of financial or non-financial support for the review, and the role of	Page 32

Section and Topic	Item #	Checklist item	Location where item is reported
		the funders or sponsors in the review.	
Competing interests	26	Declare any competing interests of review authors.	Pages 32, 33
Availability of data, code and other materials	27	Report which of the following are publicly available and where they can be found: template data collection forms; **data extracted from included studies**; data used for all analyses; analytic code; any other materials used in the review.	Page 32, Table 3

From: Page MJ, McKenzie JE, Bossuyt PM, Boutron I, Hoffmann TC, Mulrow CD, et al. The PRISMA 2020 statement: an updated guideline for reporting systematic reviews. BMJ 2021;372:n71. doi: 10.1136/bmj.n71

Supplementary Material S2. Search Strategy

EMBASE

Database: Embase Classic <1947 to 1973> Part 1 of 2, Embase <1974 to 2023 September 19>
Search Strategy:
----

1 exp puerperium/
2 ((post$ adj2 part$) or post-part$ or postpart$).tw.
3 ((post$ adj2 natal$) or post-natal$ or postnatal$).tw.
4 ((post$ adj2 birth$) or post-birth$ or postbirth$).tw.
5 ((follow$ or after) adj2 birth$).tw.
6 exp perinatal period/
7 (peri-natal$ or perinatal$).tw.
8 (peri-part$ or peripart$).tw.
9 (ante-natal$ or antenatal$).tw.
10 (ante-part$ or antepart$).tw.
11 (pre-natal$ or prenatal$).tw.
12 (pre-part$ or prepart$).tw.
13 (intra-natal$ or intranatal$).tw.
14 (intra-part$ or intrapart$).tw.
15 exp pregnancy/ (824064)
16 (pregnan$ or gestat$ or birth or obstetric$).tw.
17 exp birth/
18 parturition$.tw.
19 partus.tw.
20 puerper$.tw.
21 exp parent/
22 exp caregiver/
23 (parent$ or matern$ or patern$ or mother$ or father$ or caregiver$).tw.
24 1 or 2 or 3 or 4 or 5 or 6 or 7 or 8 or 9 or 10 or 11 or 12 or 13 or 14 or 15 or 16 or 17 or 18 or 19 or 20 or 21 or 22 or 23
25 exp mental disease/
26 ((mental$ or psych$) adj2 (health$ or illness$ or symptom$ or disorder$)).tw.
27 exp depression/
28 depress$.tw.
29 dysthymi$.tw.
30 melanchol$.tw.
31 (low adj2 mood$).tw.
32 (mood adj2 (illness$ or symptom$ or disorder$)).tw.
33 (adjustment adj2 (illness$ or symptom$ or disorder$)).tw.
34 ((affect or affective) adj2 (illness$ or symptom$ or disorder$)).tw.
35 25 or 26 or 27 or 28 or 29 or 30 or 31 or 32 or 33 or 34
36 24 and 35
37 exp postnatal depression/
38 36 or 37

39 exp immunization/
40 exp vaccine/
41 (immunis$ or immuniz$ or vaccin$ or inoculat$).tw.
42 39 or 40 or 41
43 38 and 42
44 (animal$ not human$).sh.
45 43 not 44

MEDLINE

Database: Ovid MEDLINE(R) ALL <1946 to September 18, 2023>
Search Strategy:
----
1 exp Postpartum Period/
2 ((post$ adj2 part$) or post-part$ or postpart$).tw.
3 ((post$ adj2 natal$) or post-natal$ or postnatal$).tw.
4 ((post$ adj2 birth$) or post-birth$ or postbirth$).tw.
5 ((follow$ or after) adj2 birth$).tw.
6 exp Peripartum Period/
7 (peri-natal$ or perinatal$).tw.
8 (peri-part$ or peripart$).tw.
9 (ante-natal$ or antenatal$).tw.
10 (ante-part$ or antepart$).tw.
11 (pre-natal$ or prenatal$).tw.
12 (pre-part$ or prepart$).tw.
13 (intra-natal$ or intranatal$).tw.
14 (intra-part$ or intrapart$).tw.
15 exp Pregnancy/
16 (pregnan$ or gestat$ or birth or obstetric$).tw.
17 exp Parturition/
18 parturition$.tw.
19 partus.tw.
20 puerper$.tw.
21 exp Parents/
22 exp Caregivers/
23 (parent$ or matern$ or patern$ or mother$ or father$ or caregiver$).tw.
24 1 or 2 or 3 or 4 or 5 or 6 or 7 or 8 or 9 or 10 or 11 or 12 or 13 or 14 or 15 or 16 or 17 or 18 or 19 or 20 or 21 or 22 or 23
25 exp Mental Disorders/
26 ((mental$ or psych$) adj2 (health$ or illness$ or symptom$ or disorder$)).tw.
27 exp Depression/
28 depress$.tw.
29 dysthymi$.tw.
30 melanchol$.tw.
31 (low adj2 mood$).tw.
32 (mood adj2 (illness$ or symptom$ or disorder$)).tw.
33 (adjustment adj2 (illness$ or symptom$ or disorder$)).tw.
34 ((affect or affective) adj2 (illness$ or symptom$ or disorder$)).tw.
35 25 or 26 or 27 or 28 or 29 or 30 or 31 or 32 or 33 or 34
36 24 and 35
37 exp Depression, Postpartum/
38 exp Pregnancy Complications/px [Psychology]
39 37 or 38
40 36 or 39

41 exp Immunization/
42 exp Immunization Programs/
43 exp Vaccines/
44 (immunis$ or immuniz$ or vaccin$ or inoculat$).tw.
45 41 or 42 or 43 or 44
46 40 and 45
47 exp animals/ not humans.sh.
48 46 not 47

PsycINFO

Database: APA PsycInfo <1806 to September Week 2 2023>
Search Strategy:

----

1 exp Postnatal Period/
2 ((post$ adj2 part$) or post-part$ or postpart$).tw.
3 ((post$ adj2 natal$) or post-natal$ or postnatal$).tw.
4 ((post$ adj2 birth$) or post-birth$ or postbirth$).tw.
5 ((follow$ or after) adj2 birth$).tw.
6 exp Perinatal Period/
7 (peri-natal$ or perinatal$).tw.
8 (peri-part$ or peripart$).tw.
9 (ante-natal$ or antenatal$).tw.
10 (ante-part$ or antepart$).tw.
11 (pre-natal$ or prenatal$).tw.
12 (pre-part$ or prepart$).tw.
13 (intra-natal$ or intranatal$).tw.
14 (intra-part$ or intrapart$).tw.
15 exp Pregnancy/
16 (pregnan$ or gestat$ or birth or obstetric$).tw.
17 exp Birth/
18 parturition$.tw.
19 partus.tw.
20 puerper$.tw.
21 exp Parents/
22 exp Caregivers/
23 (parent$ or matern$ or patern$ or mother$ or father$ or caregiver$).tw.
24 1 or 2 or 3 or 4 or 5 or 6 or 7 or 8 or 9 or 10 or 11 or 12 or 13 or 14 or 15 or 16 or 17 or 18 or 19 or 20 or 21 or 22 or 23
25 exp mental disorders/
26 ((mental$ or psych$) adj2 (health$ or illness$ or symptom$ or disorder$)).tw.
27 exp major depression/
28 depress$.tw.
29 dysthymi$.tw.
30 melanchol$.tw.
31 (low adj2 mood$).tw.
32 (mood adj2 (illness$ or symptom$ or disorder$)).tw.
33 (adjustment adj2 (illness$ or symptom$ or disorder$)).tw.
34 ((affect or affective) adj2 (illness$ or symptom$ or disorder$)).tw.
35 25 or 26 or 27 or 28 or 29 or 30 or 31 or 32 or 33 or 34
36 24 and 35
37 exp Postpartum Depression/
38 36 or 37
39 exp Immunization/
40 (immunis$ or immuniz$ or vaccin$ or inoculat$).tw.

41 39 or 40
42 38 and 41
43 exp animals/ not humans.sh.
44 42 not 43

SCOPUS

31 (((TITLE-ABS-KEY (((mental* OR psych*) W/2 (health* OR illness* OR symptom* OR disorder*)))) OR (TITLE-ABS-KEY (depress*)) OR (TITLE-ABS-KEY (dysthymi*)) OR (TITLE-ABS-KEY (melanchol*)) OR (TITLE-ABS-KEY ((low W/2 mood*))) OR (TITLE-ABS-KEY ((mood W/2 (illness* OR symptom* OR disorder*)))) OR (TITLE-ABS-KEY ((adjustment W/2 (illness* OR symptom* OR disorder*)))) OR (TITLE-ABS-KEY (((affect OR affective) W/2 (illness* OR symptom* OR disorder*))))) AND ((TITLE-ABS-KEY (((post* W/2 part*) OR post-part* OR postpart*))) OR (TITLE-ABS-KEY (((post* W/2 natal*) OR post-natal* OR postnatal*))) OR (TITLE-ABS-KEY (((post* W/2 birth*) OR post-birth* OR postbirth*))) OR (TITLE-ABS-KEY (((follow* OR after) W/2 birth*))) OR (TITLE-ABS-KEY ((peri-natal* OR perinatal*))) OR (TITLE-ABS-KEY ((peri-part* OR peripart*))) OR (TITLE-ABS-KEY ((ante-natal* OR antenatal*))) OR (TITLE-ABS-KEY ((ante-part* OR antepart*))) OR (TITLE-ABS-KEY ((pre-natal* OR prenatal*))) OR (TITLE-ABS-KEY ((pre-part* OR prepart*))) OR (TITLE-ABS-KEY ((intra-natal* OR intranatal*))) OR (TITLE-ABS-KEY ((intra-part* OR intrapart*))) OR (TITLE-ABS-KEY ((pregnan* OR gestat* OR birth OR obstetric*))) OR (TITLE-ABS-KEY (parturition*)) OR (TITLE-ABS-KEY (partus)) OR (TITLE-ABS-KEY (puerper*)) OR (TITLE-ABS-KEY ((parent* OR matern* OR patern* OR mother* OR father* OR caregiver*))))) AND (TITLE-ABS-KEY ((immunis* OR immuniz* OR vaccin* OR inoculat*))) AND (EXCLUDE (EXACTKEYWORD , "animal"))

30 (((TITLE-ABS-KEY (((mental* OR psych*) W/2 (health* OR illness* OR symptom* OR disorder*)))) OR (TITLE-ABS-KEY (depress*)) OR (TITLE-ABS-KEY (dysthymi*)) OR (TITLE-ABS-KEY (melanchol*)) OR (TITLE-ABS-KEY ((low W/2 mood*))) OR (TITLE-ABS-KEY ((mood W/2 (illness* OR symptom* OR disorder*)))) OR (TITLE-ABS-KEY ((adjustment W/2 (illness* OR symptom* OR disorder*)))) OR (TITLE-ABS-KEY (((affect OR affective) W/2 (illness* OR symptom* OR disorder*))))) AND ((TITLE-ABS-KEY (((post* W/2 part*) OR post-part* OR postpart*))) OR (TITLE-ABS-KEY (((post* W/2 natal*) OR post-natal* OR postnatal*))) OR (TITLE-ABS-KEY (((post* W/2 birth*) OR post-birth* OR postbirth*))) OR (TITLE-ABS-KEY (((follow* OR after) W/2 birth*))) OR (TITLE-ABS-KEY ((peri-natal* OR perinatal*))) OR (TITLE-ABS-KEY ((peri-part* OR peripart*))) OR (TITLE-ABS-KEY ((ante-natal* OR antenatal*))) OR (TITLE-ABS-KEY ((ante-part* OR antepart*))) OR (TITLE-ABS-KEY ((pre-natal* OR prenatal*))) OR (TITLE-ABS-KEY ((pre-part* OR prepart*))) OR (TITLE-ABS-KEY ((intra-natal* OR intranatal*))) OR (TITLE-ABS-KEY ((intra-part* OR intrapart*))) OR (TITLE-ABS-KEY ((pregnan* OR gestat* OR birth OR obstetric*))) OR (TITLE-ABS-KEY (parturition*)) OR (TITLE-ABS-KEY (partus)) OR (TITLE-ABS-KEY (puerper*)) OR (TITLE-ABS-KEY ((parent* OR matern* OR patern* OR mother* OR father* OR caregiver*))))) AND (TITLE-ABS-KEY ((immunis* OR immuniz* OR vaccin* OR inoculat*)))

29 TITLE-ABS-KEY ((immunis* OR immuniz* OR vaccin* OR inoculat*))

28 ((TITLE-ABS-KEY (((mental* OR psych*) W/2 (health* OR illness* OR symptom* OR disorder*)))) OR (TITLE-ABS-KEY (depress*)) OR (TITLE-ABS-KEY (dysthymi*)) OR (TITLE-ABS-KEY (melanchol*)) OR (TITLE-ABS-KEY ((low W/2 mood*))) OR (TITLE-ABS-KEY ((mood W/2 (illness* OR symptom* OR disorder*)))) OR (TITLE-ABS-KEY ((adjustment W/2 (illness* OR symptom* OR disorder*)))) OR (TITLE-ABS-KEY (((affect OR affective) W/2 (illness* OR symptom* OR disorder*))))) AND ((TITLE-ABS-KEY (((post* W/2 part*) OR post-part* OR postpart*))) OR (TITLE-ABS-KEY (((post* W/2 natal*) OR post-natal* OR postnatal*))) OR (TITLE-ABS-KEY (((post* W/2 birth*) OR post-birth* OR postbirth*))) OR (TITLE-ABS-KEY (((follow* OR after) W/2 birth*))) OR (TITLE-ABS-KEY ((peri-natal* OR perinatal*))) OR (TITLE-ABS-KEY ((peri-part* OR peripart*))) OR (TITLE-ABS-KEY ((ante-natal* OR antenatal*))) OR (TITLE-ABS-KEY ((ante-part* OR antepart*))) OR (TITLE-ABS-KEY ((pre-natal* OR prenatal*))) OR (TITLE-ABS-KEY ((pre-part* OR prepart*))) OR (TITLE-ABS-KEY ((intra-natal* OR intranatal*))) OR (TITLE-ABS-KEY ((intra-part* OR intrapart*))) OR (TITLE-ABS-KEY ((pregnan* OR gestat* OR birth OR obstetric*))) OR (TITLE-ABS-KEY (parturition*)) OR (TITLE-ABS-KEY (partus)) OR (TITLE-ABS-KEY (puerper*)) OR (TITLE-ABS-KEY ((parent* OR matern* OR patern* OR mother* OR father* OR caregiver*))))

27 (TITLE-ABS-KEY (((mental* OR psych*) W/2 (health* OR illness* OR symptom* OR disorder*)))) OR (TITLE-ABS-KEY (depress*)) OR (TITLE-ABS-KEY (dysthymi*)) OR (TITLE-ABS-KEY (melanchol*)) OR (TITLE-ABS-KEY ((low W/2 mood*))) OR (TITLE-ABS-KEY ((mood W/2 (illness* OR symptom* OR disorder*)))) OR (TITLE-ABS-KEY ((adjustment W/2 (illness* OR symptom* OR disorder*)))) OR (TITLE-ABS-KEY (((affect OR affective) W/2 (illness* OR symptom* OR disorder*))))

26 TITLE-ABS-KEY (((affect OR affective) W/2 (illness* OR symptom* OR disorder*)))

25 TITLE-ABS-KEY ((adjustment W/2 (illness* OR symptom* OR disorder*)))

24 TITLE-ABS-KEY ((mood W/2 (illness* OR symptom* OR disorder*)))

23 TITLE-ABS-KEY ((low W/2 mood*))

22 TITLE-ABS-KEY (melanchol*)

21 TITLE-ABS-KEY (dysthymi*)

20 TITLE-ABS-KEY (depress*)

19 TITLE-ABS-KEY (((mental* OR psych*) W/2 (health* OR illness* OR symptom* OR disorder*)))

18 (TITLE-ABS-KEY (((post* W/2 part*) OR post-part* OR postpart*))) OR (TITLE-ABS-KEY (((post* W/2 natal*) OR post-natal* OR postnatal*))) OR (TITLE-ABS-KEY (((post* W/2 birth*) OR post-birth* OR postbirth*))) OR (TITLE-ABS-KEY (((follow* OR after) W/2 birth*))) OR (TITLE-ABS-KEY ((peri-natal* OR perinatal*))) OR (TITLE-ABS-KEY ((peri-part* OR peripart*))) OR (TITLE-ABS-KEY ((ante-natal* OR antenatal*))) OR (TITLE-ABS-KEY ((ante-part* OR antepart*))) OR (TITLE-ABS-KEY ((pre-natal* OR prenatal*))) OR (TITLE-ABS-KEY ((pre-part* OR prepart*))) OR (TITLE-ABS-KEY ((intra-natal* OR intranatal*))) OR (TITLE-ABS-KEY ((intra-part* OR intrapart*))) OR (TITLE-ABS-KEY ((pregnan* OR gestat* OR birth OR obstetric*))) OR (TITLE-ABS-KEY (parturition*)) OR (TITLE-ABS-KEY (partus)) OR (TITLE-ABS-KEY (puerper*)) OR (TITLE-ABS-KEY ((parent* OR matern* OR patern* OR mother* OR father* OR caregiver*)))

17 TITLE-ABS-KEY ((parent* OR matern* OR patern* OR mother* OR father* OR caregiver*))

16 TITLE-ABS-KEY (puerper*)

15 TITLE-ABS-KEY (partus)

14 TITLE-ABS-KEY (parturition*)

13 TITLE-ABS-KEY ((pregnan* OR gestat* OR birth OR obstetric*))

12 TITLE-ABS-KEY ((intra-part* OR intrapart*))

11 TITLE-ABS-KEY ((intra-natal* OR intranatal*))

10 TITLE-ABS-KEY ((pre-part* OR prepart*))

9 TITLE-ABS-KEY ((pre-natal* OR prenatal*))

8 TITLE-ABS-KEY ((ante-part* OR antepart*))

7 TITLE-ABS-KEY ((ante-natal* OR antenatal*))

6 TITLE-ABS-KEY ((peri-part* OR peripart*))

5 TITLE-ABS-KEY ((peri-natal* OR perinatal*))

4 TITLE-ABS-KEY (((follow* OR after) W/2 birth*))

3 TITLE-ABS-KEY (((post* W/2 birth*) OR post-birth* OR postbirth*))

2 TITLE-ABS-KEY (((post* W/2 natal*) OR post-natal* OR postnatal*))

1 TITLE-ABS-KEY (((post* W/2 part*) OR post-part* OR postpart*))

CINAHL via EBSCO

Search ID#	Search Terms
S51	S45 NOT S50
S50	S48 NOT S49
S49	(MH "Human")
S48	S46 OR S47
S47	(MH "Animal Studies")
S46	(MH "Animals+")
S45	S39 AND S44
S44	S40 OR S41 OR S42 OR S43
S43	(immunis* OR immuniz* OR vaccin* OR inoculat*)
S42	(MH "Vaccines+")
S41	(MH "Immunization Programs")
S40	(MH "Immunization+")
S39	S35 OR S38
S38	S36 OR S37
S37	(MH "Pregnancy Complications+/PF")
S36	(MH "Depression, Postpartum")
S35	S23 AND S34

S34	S24 OR S25 OR S26 OR S27 OR S28 OR S29 OR S30 OR S31 OR S32 OR S33
S33	((affect OR affective) W2 (illness* OR symptom* OR disorder*))
S32	(adjustment W2 (illness* OR symptom* OR disorder*))
S31	(mood W2 (illness* OR symptom* OR disorder*))
S30	(low W2 mood*)
S29	melanchol*
S28	dysthymi*
S27	depress*
S26	(MH "Depression+")
S25	((mental* OR psych*) W2 (health* OR illness* OR symptom* OR disorder*))
S24	(MH "Mental Disorders+")
S23	S1 OR S2 OR S3 OR S4 OR S5 OR S6 OR S7 OR S8 OR S9 OR S10 OR S11 OR S12 OR S13 OR S14 OR S15 OR S16 OR S17 OR S18 OR S19 OR S20 OR S21 OR S22
S22	(parent* OR matern* OR patern* OR mother* OR father* OR caregiver*)
S21	(MH "Caregivers")
S20	(MH "Parents+")
S19	puerper*
S18	partus

S17	parturition*
S16	(pregnan* OR gestat* OR birth OR obstetric*)
S15	(MH "Pregnancy+")
S14	(intra-part* OR intrapart*)
S13	(intra-natal* OR intranatal*)
S12	(pre-part* OR prepart*)
S11	(pre-natal* OR prenatal*)
S10	(ante-part* OR antepart*)
S9	(ante-natal* OR antenatal*)
S8	(peri-part* OR peripart*)
S7	(peri-natal* OR perinatal*)
S6	(MH "Perinatal Period")
S5	((follow* OR after) W2 birth*)
S4	((post* W2 birth*) OR post-birth* OR postbirth*)
S3	((post* W2 natal*) OR post-natal* OR postnatal*)
S2	((post* W2 part*) OR post-part* OR postpart*)
S1	(MH "Postnatal Period+")

Web of Science Core Collection

Science Citation Index Expanded (SCI-EXPANDED)--1900-present

Social Sciences Citation Index (SSCI)--1956-present

Social Sciences Citation Index (SSCI)--1956-present

Arts & Humanities Citation Index (AHCI)--1975-present

Arts & Humanities Citation Index (AHCI)--1975-present

Conference Proceedings Citation Index – Science (CPCI-S)--1990-present

Conference Proceedings Citation Index – Science (CPCI-S)--1990-present

Conference Proceedings Citation Index – Social Science & Humanities (CPCI-SSH)--1990-present

Conference Proceedings Citation Index – Social Science & Humanities (CPCI-SSH)--1990-present

Book Citation Index – Science (BKCI-S)--2005-present

Book Citation Index – Science (BKCI-S)--2005-present

Book Citation Index – Social Sciences & Humanities (BKCI-SSH)--2005-present

Book Citation Index – Social Sciences & Humanities (BKCI-SSH)--2005-present

Emerging Sources Citation Index (ESCI)--2005-present

Emerging Sources Citation Index (ESCI)--2005-present

Current Chemical Reactions (CCR-EXPANDED)--1985-present

Current Chemical Reactions (CCR-EXPANDED)--1985-present

Index Chemicus (IC)--1993-present

30 #29 AND #28

29 immunis* OR immuniz* OR vaccin* OR inoculat* (Topic)

28 #27 AND #18

27 #19 OR #20 OR #21 OR #22 OR #23 OR #24 OR #25 OR #26

26 (affect OR affective) NEAR/2 (illness* OR symptom* OR disorder*) (Topic)

25 (adjustment NEAR/2 (illness* OR symptom* OR disorder*)) (Topic)

24 (mood NEAR/2 (illness* OR symptom* OR disorder*)) (Topic)

23 low NEAR/2 mood* (Topic)

22 melanchol* (Topic)

21 dysthymi* (Topic)

20 depress* (Topic)

19 (mental* OR psych*) NEAR/2 (health* OR illness* OR symptom* OR disorder*) (Topic)

18 #1 OR #2 OR #3 OR #4 OR #5 OR #6 OR #7 OR #8 OR #9 OR #10 OR #11 OR #12 OR #13 OR #14 OR #15 OR #16 OR #17

17 parent* OR matern* OR patern* OR mother* OR father* OR caregiver* (Topic)

16 puerper* (Topic)

15 partus (Topic)

14 parturition* (Topic)

13 pregnan* OR gestat* OR birth OR obstetric* (Topic)

12 intra-part* OR intrapart* (Topic)

11 intra-natal* OR intranatal* (Topic)

10 pre-part* OR prepart* (Topic)

9 pre-natal* OR prenatal* (Topic)

8 ante-part* OR antepart* (Topic)

7 ante-natal* OR antenatal* (Topic)

6 peri-part* OR peripart* (Topic)

5 peri-natal* OR perinatal* (Topic)

4 ((follow* OR after) NEAR/2 birth*) (Topic)

3 ((post* NEAR/2 birth*) OR post-birth* OR postbirth*) (Topic)

2 ((post* NEAR/2 natal*) OR post-natal* OR postnatal*) (Topic)

1 ((post* NEAR/2 part*) OR post-part* OR postpart*) (Topic)

LILACS

((post-part* OR postpart* OR "post* natal*" OR post-natal* OR postnatal* OR "post* birth*" OR post-birth* OR postbirth* OR "follow* birth*" OR "after birth*" OR peri-part* OR peripart* OR peri-natal* OR perinatal OR intra-part* OR intrapart* OR intra-natal* OR intranatal* OR pre-part* OR prepart* OR pre-natal* OR prenatal* OR ante-part* OR antepart* OR ante-natal* OR antenatal* OR pregnan* OR gestat* OR birth OR obstetric* OR puerper* OR partus OR parturition* OR parent* OR matern* OR patern* OR mother* OR father* OR caregiver*)) AND (depress* OR dysthymi* OR melanchol* OR "low mood*") AND ((immunis* OR immuniz* OR vaccin* OR inoculat*))

Sociological Abstracts via ProQuest, 1952-current

Set#: S1
Searched for: noft(post-part* OR postpart* OR "post* natal*" OR post-natal* OR postnatal* OR "post* birth*" OR post-birth* OR postbirth* OR "follow* birth*" OR "after birth*" OR peri-part* OR peripart* OR peri-natal* OR perinatal OR intra-part* OR intrapart* OR intra-natal* OR intranatal* OR pre-part* OR prepart* OR pre-natal* OR prenatal* OR ante-part* OR antepart* OR ante-natal* OR antenatal* OR pregnan* OR gestat* OR birth OR obstetric* OR puerper* OR partus OR parturition* OR parent* OR matern* OR patern* OR mother* OR father* OR caregiver*) AND noft(depress* OR dysthymi* OR melanchol* OR "low mood*" OR "mental* health*" OR "mental* illness*" OR "mental* symptom*" OR "mental* disorder*" OR "psych* health*" OR "psych* illness*" OR "psych* symptom*" OR "psych* disorder*" OR "adjustment illness*" OR "adjustment symptom*" OR "adjustment disorder*" OR "mood illness*" OR "mood symptom*" OR "mood disorder*") AND noft(immunis* OR immuniz* OR vaccin* OR inoculat*) AND PEER(yes)

(NOFT = all fields except full text)
